# Emerging Technologies to Enable Sustainable Controlled Environment Agriculture in the Extreme Environments of Middle East-North Africa Coastal Regions

**DOI:** 10.3389/fpls.2020.00801

**Published:** 2020-07-02

**Authors:** Ryan M. Lefers, Mark Tester, Kyle J. Lauersen

**Affiliations:** ^1^Biological and Environmental Science and Engineering Division, King Abdullah University of Science and Technology, Thuwal, Saudi Arabia; ^2^Water Desalination and Reuse Center, Biological and Environmental Science and Engineering Division, King Abdullah University of Science and Technology, Thuwal, Saudi Arabia; ^3^Center for Desert Agriculture, Biological and Environmental Science and Engineering Division, King Abdullah University of Science and Technology, Thuwal, Saudi Arabia; ^4^Texas AgriLife Research and Extension Center at Dallas, Texas A&M University, Dallas, TX, Unites States

**Keywords:** infrared solar, evaporative desiccant cooling, sustainability, combinatorial farming, algal biotechnology, salt water agriculture

## Abstract

Despite global shifts in attitudes toward sustainability and increasing awareness of human impact on the environment, projected population growth and climate change require technological adaptations to ensure food and resource security at a global scale. Although desert areas have long been proposed as ideal sites for solar electricity generation, only recently have efforts shifted toward development of specialized and regionally focused agriculture in these extreme environments. In coastal regions of the Middle East and North Africa (MENA), the most abundant resources are consistent intense sunlight and saline sea water. MENA coastal regions hold incredible untapped potential for agriculture driven by the combination of key emerging technologies in future greenhouse concepts: transparent infrared collecting solar panels and low energy salt water cooling. These technologies can be combined to create greenhouses that drive regionally relevant agriculture in this extreme environment, especially when the target crops are salt-tolerant plants and algal biomass. Future controlled environment agriculture concepts will not compete for municipal fresh water and can be readily integrated into local human/livestock/fisheries food chains. With strategic technological implementation, marginal lands in these environments could participate in production of biomass, sustainable energy generation, and the circular carbon economy. The goal of this perspective is to reframe the idea of these environments as extreme, to having incredible untapped development potential.

## Article

Global changes in mean surface temperatures are driving increased extreme environmental events which are beyond the tolerance of traditional agricultural practices, creating concerns of food insecurity (Hansen et al., 2012; Rhines and Huybers, 2013; Stone et al., 2013; Sutton et al., 2015; Bathiany et al., 2018; Spinoni et al., 2018). A logical step toward increasing agricultural yield predictability is to move toward contained agriculture concepts like greenhouses. Glasshouses, or other controlled environment agriculture (CEA) structures, are traditionally applied to extend growth and cultivation periods in cooler climates, reducing frost damage to crops or for specialty/ornamental plant cultivation. CEA enables expansion of horticulture into non-traditional environments and marginal lands, benefits that can contribute to increasing output and food security. The application of CEA in hot or desert environments has been less common than in temperate climates or higher latitudes, owing to the energy required for cooling these structures. The Middle East and North Africa (MENA) regions have some of most extreme desert climates in the world, with high average annual temperatures and very low precipitation (Beck et al., 2018). However, in coastal regions, these environments are rich in two key resources: consistent solar radiation and sea water. Development of technological solutions which work with these resources in this coastal climate to drive sustainable agricultural practices can assist the region to meaningfully contribute to the global bio-economy and local food security.

The consistent solar radiation and strong daily winds of the MENA region can readily be used for sustainable electricity generation by traditional photovoltaics and wind turbines. The global horizontal irradiance (GHI) over the Arabian Peninsula shows significant seasonal variations with an annual mean ranging between 0.75 and 1.06 kW m^–2^, with higher values over the northwestern region (Dasari et al., 2019). The wind resources over Saudi Arabia exhibit strong spatial variations, with high annual mean wind power density at 80 m height over the northern Red Sea (>0.8 kW m^–2^), indicating the capacity for these technologies to support local CEA efforts (Langodan et al., 2016). However, for CEA systems to be practical in hot environments, technological solutions are required to minimize operational energy needs, especially for cooling. The combination of low-energy cooling systems with heat-reducing energy-generating technologies will improve overall CEA operational efficiencies and enable their implementation in MENA coastal regions. These systems become even more attractive when mixed salt-tolerant plant species and algal biomass cultivation are combined in high density cultivation concepts to reduce freshwater requirements. Intensive novel crop combinations in future CEA concepts may provide practical regional solutions for food generation while contributing to carbon capture and cycling. This work seeks to shift the perception of coastal MENA regions from extreme and inhospitable environments to locations with ample resources in the form of sunlight and sea water which could be developed into global agricultural powerhouses. These two resources can be combined to drive a regional agricultural revolution when appropriate technologies are implemented with strategic species selection.

Traditional agriculture in MENA regions includes cultivation of date palms (Erskine et al., 2004), and some species of plants which are tolerant to local environment such as *Salvadora* (miswak). Recent developments have seen increased efforts toward aquaculture farming (i.e., NAQUA farms, KSA)^1^. In addition, there are increasing outdoor horticultural efforts in various regions, with Saudi Arabia and Oman being the largest participants (Erskine et al., 2004; Noorka and Heslop-Harrison, 2015). Agriculture in these environments requires large inputs of freshwater, which is dominated by small amounts from desalination and significant extractions from aquifers, the latter being largely unsustainable (Gleeson et al., 2012). Indeed, 80% of freshwater resources in the Gulf region are used for agricultural practices (Erskine et al., 2004; Noorka and Heslop-Harrison, 2015). Hydroponic or CEA systems like those which are practiced in greenhouses use a fraction of the freshwater and waste less fertilizers than field agriculture. Furthermore, there is a sound economic and environmental case for CEA in this region for a large number of vegetables, and even some fresh fruits. We estimate that up to 70% (on a fresh weight basis) of fresh fruits and vegetables could be economically grown locally, primarily facilitated by use of CEA, with an overall lower environmental footprint than from the use of imported food. The contribution of implementation of this practice to local food security cannot be over-stated.

**FIGURE 1:**
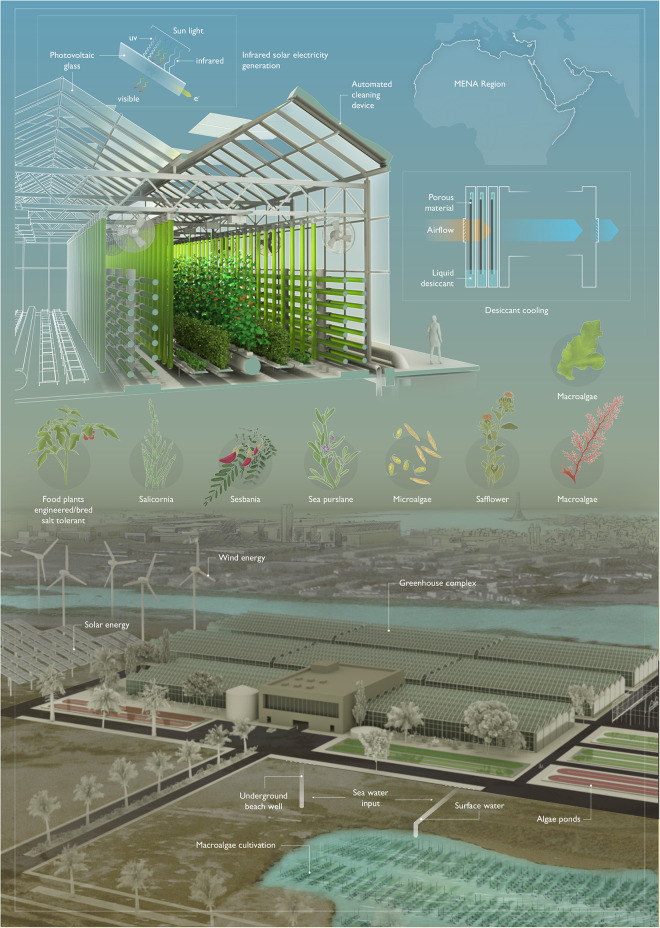
A low-energy glasshouse concept for future agriculture in coastal MENA regions. Future CEA greenhouses will combine infrared solar energy capture and desiccant cooling technologies to create stable contained environments for horticulture in extreme desert coastal environments. Infrared harvesting transparent solar panels allow photosynthetic active radiation (visible spectrum) to penetrate transparent glass surfaces to enable photosynthesis while simultaneously reducing the heating effect. Passive cooling can be achieved by passing hot external humid air through highly saline liquid desiccant solutions in porous matrices which adsorb air moisture, releasing dry, cooler air due to the vapor pressure difference. Coupling these technologies with high density hydroponic cultivation concepts and combined algae photobioreactors (green tubes) will maximize biomass productivity in these systems using seawater as cultivation medium. Macroalgae farming may also be an attractive addition to these concepts and can be coupled in managed pools on land or in the surrounding sea for nutrient removal and intensified biomass production. Plants which are naturally tolerant or those bred/engineered for salinity tolerance (pictured) can be cultivated with locally available sea water resources to minimize fresh-water requirements. Sustainable energy generation by traditional photovoltaics and wind turbines can be combined to support the energy requirements of these facilities.

Two key technologies can enable low-energy greenhouses in hot coastal environments: these are efficient organic transparent infrared solar panels and liquid desiccant-based cooling (Figure 1). Newly reported advances in efficient infrared organic solar panels have shown key efficiency advances in the capture of latent heat energy to generate electricity (Song et al., 2018). Using a blend of 4% 1-chloronaphthalene as a solvent and the narrow-band-gap non-fullerene acceptor IEICO-4F, a thin material which absorbs maximally at 900 nm was shown to generate 26.8 mA cm^–2^ with photo conversion efficiencies of 12.8% (Song et al., 2018). The narrow bandgap of IEICO-4F allows penetration of photosynthetic wavelengths of light (400–700 nm) unlike commercial silicon technology and is suitable for roll-to-roll production processes. This technology will allow the surfaces of greenhouses and windows to generate electricity, while simultaneously serving as a transparent enclosure to enable plant and algal growth. Capture of infrared energy will also reduce heating effects of sun-light within the CEA structure, thereby reducing the energy required for cooling. Surfaces in MENA regions are also prone to dust buildup and advances in mechanical automated dusting devices can now be implemented to ensure efficient operation of photovoltaics and glasshouse surfaces^2^.

Future CEA designs can be even more energy efficient when combined with low-energy air cooling technologies, especially liquid desiccant (Bettahalli et al., 2016; Lefers et al., 2016) or evaporative cooling (Chua et al., 1999; Lefers et al., 2018b; Shahzad et al., 2019). Liquid desiccant cooling relies on highly saline solutions that capture moisture from hot humid air and remove latent heat as moisture is absorbed by the desiccant. Moisture removal from humid air results in a pronounced cooling effect in the air passed through these structures as latent heat is removed (Lefers et al., 2016). Liquid desiccant systems can be further combined with sea water evaporative cooling systems to substitute the latent cooling achieved by the liquid desiccant system pending crop needs. Although sites will not be independent from municipal freshwater use, vacuum regeneration of the liquid desiccant can be applied to yield additional freshwater as a by-product, which can support horticulture within the glasshouse (Lefers et al., 2018a). It is likely that future CEA concepts will be coupled to desalination efforts near local municipalities to supply the freshwater needed for human as well as some plant use. Evaporative cooling technologies utilizing sea water could also provide a lower energy solution and can be coupled to on site desalination plants, such as reverse osmosis and adsorption desalination, to provide more abundant freshwater resources without increasing energy demands and reducing the amount of brine from desalination processes (Ng et al., 2013). The combination of infrared solar cells on greenhouse surfaces with low-energy cooling and desalination technologies provides the preconditions for design of energy efficient and sustainable structures to enable CEA concepts in hot environments. In addition to water-use and temperature control, other challenges to address for CEA in MENA coastal regions include brine management, wastewater reuse, automation, and sustainable sourcing of plant nutrients. Needless to say, for widespread adoption of innovations, demonstration of cost-effectiveness at scale is essential. Our preliminary studies suggest that the modest increases in CapEx for such CEA systems are partially offset by reduced OpEx and reduced transportation. Overall, the extra costs incurred by use of salt water are very modest and do not have a significant effect on the overall business case for such greenhouses.

In addition to structural and technological considerations, selection of appropriate saline tolerant or drought resistant plant species is another key contributing factor in the success of future CEA in MENA coastal regions. New initiatives in the Gulf region are underway to promote desert agriculture using salt tolerant plant species such as *Salicornia*, the group of plants collectively known as sea purslane, *Chenopodium quinoa* (quinoa), miswak, *Sesbania*, *Triticale*, and *Carthamus* (safflower)^3^. The efficiency of water use could also be improved if appropriate saline-tolerant crops are cultivated in full or partial sea-water. Coastal regions are especially valuable in this context as beach wells can provide abundant naturally filtered, thermally consistent, saline water. Combination of freshwater plant species with saline-tolerant crops and algae in combined high-density cultivation concepts has the potential to generate robust biomass production processes in smaller land areas than by traditional field agriculture while using less overall freshwater resources. Plants such as grasses or quinoa are able to be cultivated in marginal lands and arid outdoor environments, and greenhouses are not necessary for their enhanced agricultural production. However, wild, bred or engineered food crops, like recently described saline tolerant tomato varieties (Pailles et al., 2020) and edible greens, will be well suited to high density cultivation in contained greenhouses in MENA coastal regions. The role of genetically modified (GM) crops in this region is a large topic which could be its own article, permissions for various crops have been granted in Egypt, Turkey, Sudan, Iran, and Pakistan^4^. However, the use of GMs is completely prohibited at the time of writing in countries like Saudi Arabia. CEA would allow some amounts of containment and minimize environmental risks/concerns over GM crops, however, improved hardiness would be less important in these controlled environments. GM may be interesting for nutritional improvements of crops grown in CEA, such as increased anthocyanin or phenylpropanoid contents of tomatoes (Butelli et al., 2008; Zhang et al., 2015). Modified traits may be more valuable for microalgal cultivation where increases in lipid content (Ajjawi et al., 2017) or novel traits (Lauersen, 2019; Fabris et al., 2020) are highly desired and add value to the biomass. Ongoing difficulties with consumer acceptance of GM organisms and complex country specific regulatory constraints makes widespread deployment of transgenics unlikely for the foreseeable future, so we do not discuss these issues further here. We also limit the discussion of field agriculture as our work focuses on future CEA. Additional efforts in improving rhizobial interactions and desert-probiotics for crops grown in harsh environments are also steadily developing, with promising results for encouraging heat tolerance and desiccation resistance under outdoor conditions (Bang et al., 2018; Daur et al., 2018; Eida et al., 2018). These efforts can serve as a roadmap for encouraging low-water use in field agriculture for some plants that are grown outdoors in harsh environments (Saad et al., 2020). Neo-domestication of other thermo and saline tolerant plant species as well as selective breeding/engineering could potentially increase productivities of these cultivation concepts and work is accelerating in this field (Lemmon et al., 2018; Li et al., 2018; Dawson et al., 2019; Fernie and Yan, 2019; Shan-e-Ali Zaidi et al., 2019).

In future salt-water driven CEA concepts, it is likely that salt-tolerant and fresh-water cultivars will be alternated to provide balanced supplies of both types of plants for food/feed and minimize fresh water demands. Potential exists here for the introduction of non-traditional agriculture in the form of combinatorial cultivation concepts that integrate algal photobioreactors with higher plant hydroponics. Algae are rapidly growing photosynthetic organisms that can add increased productivity to the CEA system using full sea water as culture medium. Cultivation of algae is practiced both indoors and outdoors, with greenhouses providing improved environmental control as with higher plants (Posten, 2009). Integration of algal photobioreactors into future high-density CEA concepts could provide continual biomass generation for a range of applications and enhance areal carbon turnover rates (Lehr and Posten, 2009; Posten, 2009). Algal biomass can be used in aquaculture, animal feed, bioplastics and cosmetics, or to generate environmentally friendly replacements for plant-based oils (Radmer, 1996; Priyadarshani and Rath, 2012; Gangl et al., 2015; Chen et al., 2019). CEAs that include algal cultivation could act as sustainable sources for the natural inputs to greater bio-based industries, as algal cultivation can provide consistent biomass production at high turnover rates. Some reports even indicate co-cultivation of green algae like *Chlorella* and *Scenedesmus* together with the roots of higher plants in hydroponic systems has a dual benefit for both organisms which share growth promoting factors (Zhang et al., 2017; Barone et al., 2019). No work has yet been performed on combinatorial agriculture with algae and salt-water tolerant plants in controlled settings, which may be a new avenue for bio-fortification in greenhouse concepts. Industrial scale cultivation of marine algal strains such as *Nannochloropsis*, *Dunaliella*, and *Phaeodactylum* are already practiced in many locations globally both in CEA and outdoor cultivation (Laurens, 2017). In the MENA region there is potential to even further develop local strains of interest, for example, a recently described halotolerant *Chloroidium* sp. was isolated in the United Arab Emirates that has similar triacylglycerol profile to palm oil (Nelson et al., 2017). Its intensified cultivation could reduce global impact of palm agriculture as a sustainable alternative. Macroalgae may also be integrated as part of auxiliary value additions to coastal CEA concepts as they can be cultivated near shore or in pond units. Promising productivities in small scale for marine macroalgae *Asparagopsis armata* and *Ulva rigida* have been reported, and pending appropriate water flow rates, can serve as an effective biofilter to capture excess nutrients (nitrogen and phosphorous) in the form of valuable biomass (Mata et al., 2010). Very little work has been done to characterize macroalgae from the MENA region, with some studies of broad population dynamics now emerging (Geraldi et al., 2019; Ortega et al., 2019). It is likely that local bioprospecting will yield further species of interest which are adapted to regional climate conditions and can contribute to enhancing the productivities of coastal CEA hubs.

The circular carbon economy (CCE) is a concept which seeks to capture and capitalize on waste carbon which is otherwise lost to the atmosphere, usually in the form of carbon dioxide (CO_2_) and reuse it in a cyclic fashion to minimize the environmental impacts of human activities (Stahel, 2016). This practice is of special importance to the MENA region as global economic and social trends look to the post-oil economies of the future. Plants and algae conduct light driven photosynthesis to generate cellular energy, and through the reactions of the Calvin-Benson-Bassham cycle are able to fix CO_2_ into organic sugars for growth. Photosynthesis-based fixation and cycling of CO_2_ to biomass is one part of the greater spectrum of currently developing carbon capture and reuse technologies which are of interest for the development of the CCE. Plant and algal biomass represent a 1.83 weight ratio of fixed CO_2_ per unit of biomass (Chisti, 2007). This ratio improves if the biomass is lipid or carbohydrate rich and offers a direct biological route from waste carbon to valuable bio-products. Plant and algal biomass, therefore, are incredible feedstocks for sustainable CCE practices as they represent carbon captured from the atmosphere that can be reused as physical commodities. Intensified CEA concepts which emphasize high biomass productivity in marginal lands will contribute significantly to CCE practices and provide a sustainable source of biological materials for various industries.

## Conclusion and Outlook

Although greenhouses are not a new concept, emerging technologies now enable energy efficient and profitable implementation of CEA in extreme desert environments. Low-energy cooling and enhanced energy generation/temperature reduction by transparent infrared harvesting solar cells can be combined to create energy efficient greenhouses primed for future agriculture concepts on marginal coastal lands of the MENA region. The combination of high density hydroponic saline horticulture and algal cultivation can minimize impacts on freshwater water resources and maximize carbon cycling. The increased efficiency of these greenhouses can improve agricultural efforts in the MENA region, while contributing to food security and encouraging development of the CCE. It remains to be seen whether regulatory control and growing demand for locally sourced crops will enable MENA coastal regions to become hubs of future innovative agricultural practices.

## Author Contributions

All authors listed have made a substantial, direct and intellectual contribution to the work, and approved it for publication.

## Conflict of Interest

MT and RL are co-founders of Red Sea Farms. The remaining author declares that the research was conducted in the absence of any commercial or financial relationships that could be construed as a potential conflict of interest.

## Footnotes

1 http://www.naqua.com.sa

2 http://www.nomaddesertsolar.com/

3 https://www.biosaline.org

4 http://www.isaaa.org/gmapprovaldatabase/
